# Preparation and Evaluation of Buccoadhesive Films of Atenolol

**DOI:** 10.4103/0250-474X.41451

**Published:** 2008

**Authors:** B. K. Satishbabu, B. P. Srinivasan

**Affiliations:** Delhi Institute of Pharmaceutical Sciences and Research, Sector 3, Pushpvihar, New Delhi-110 017, India

**Keywords:** Mucoadhesion, buccoadhesive films, bilaminated films, atenolol

## Abstract

This paper describes the preparation of new bilayered device comprising a drug containing mucoadhesive layer and a drug free backing layer. Bilaminated films were produced by a casting/ solvent evaporation technique. The mucoadhesive layer was composed of mixture of drug and sodium alginate with or without carbopol 934 P, and backing layer was made of ethyl cellulose. The double layer structure design was expected to provide drug delivery in a unidirectional fashion to the mucosa and avoid loss of drug due to wash out with saliva. The fabricated films were subjected to *in vitro* drug release, *in vitro* permeation through porcine buccal mucosa. The bilayered films were also evaluated for mucoadhesive strength, mucoadhesive time, folding endurance, hydration studies and tensile strength.

The buccal route, as an alternative to other traditional methods of systemic drug administration, is a subject of growing interest because of its numerous advantages 1,2. It is well known that the absorption of therapeutic compounds from the oral mucosa provides a direct entry of the drug into the systemic circulation, therefore, avoiding the first pass hepatic metabolism and gastrointestinal drug degradation, which is associated with oral administration. The oral cavity is easily accessible for self medication and, hence is well accepted by patients, and is safe, since device can be easily administered and even removed from the site of application, stopping the input of drug whenever desired.

A variety of drugs have been shown to be absorbed through the oral mucosa, following administration using solutions or conventional tablets or capsules. However, the conventional buccal dosage forms show two main disadvantages. Due to involuntary swallowing of the dosage form itself or a part of it and continuous dilution of the dissolved drug by the salivary flow, an important part of the drug may not be available for absorption and they do not allow drinking and eating or speaking. Therefore, their administration is restricted to short periods of time and, consequently, controlled drug release is not within the scope of such formulations 3. From a technological point of view, an ideal buccal dosage form must have three properties; It must maintains its position in the mouth for a few hours, release the drug in a controlled fashion and provide drug release in a unidirectional way towards the mucosa. In the present study; we chose the natural polymer sodium alginate and hydrophilic mucoadhesive polymer carbopol 934 P. The daily salivary volume secreted in humans is between 0.5 and 2 *l*^4^, which is sufficient to hydrate oral mucosal dosage forms. This water rich environment of the oral cavity is the main reason behind the selection of hydrophilic polymeric matrices as vehicles for this study.

## MATERIALS AND METHODS

The following chemicals were obtained from different sources and used as received. Sodium alginate (Protonal LF 240 D) and atenolol were gift sample from M/s. Ranbaxy; Carbopol 934P was obtained from B. F. Goodrich; ethylcellulose (EC; Ethocel 10, standard premium) from Dow chemicals; glycerin and dibutylphthalte (DBP) from CDH New Delhi. All other regents were analytical grade; double distilled water was used throughout.

### Preparation of bilayered films:

Bilaminated films were produced by a casting/ solvent evaporation technique using different combinations of polymers as shown in Table 1. The backing membrane was prepared by dissolving ethyl cellulose (5%) in mixture of acetone and isopropyl alcohol (65:35), and 20 % dry weight of polymer of dibutylphthalte as plasticizer. The plasticized ethyl cellulose solution was poured in to a 63 mm glass mould on level surface and solvent was allowed to evaporate at controlled rate by covering the mould with inverted glass funnel, to avoid blistering effect on dried films. The mucoadhesive layer was prepared using different combinations of polymers as shown in Table 1. Both sodium alginate and other polymers were dissolved in double distilled water, after complete dissolution was over the drug was added. 10% Glycerin was added as plasticizer. The plasticized polymeric solution was poured in to mould containing a backing membrane and oven dried at temp of 40° for overnight, the dried films were kept in desiccators till further use. The thickness of the prepared films were measured using screw gauge at six different areas And thickness of the films is given in Table 2.

**TABLE 1:** TABLE SHOWING FORMULATION OF SODIUM ALGINATE MUCOADHESIVE BUCCAL FILMS

Product code	SA I	SA II	SA III
Sodium alginate	1000 mg	900 mg	500 mg
Carbopol934P	0	100 mg	500 mg
Atenolol	100 mg	100 mg	100 mg
Glycerin	10%	10%	10%
Distilled water	40 ml	40 ml	40 ml

**TABLE 2:** IMPORTANT PHYSCAL AND MUCOADHESIVE PARAMETERS OF SODIUM ALGINATE FILMS

Product code	Bioadhesive strength (g)	Force of adhesion (N)	Bond strength (N/m^2^)	Surface pH	Hydration rate	Permeability co efficient	Water vapor transmission rate	Tensile strength (g/cm^2^)	Thickness (mm)
SA I	27.7	0.27	154.1	6.9	0.43	0.088	0.09	113.66	0.42
SA II	25.5	0.25	141.7	6.7	0.95	0.066	0.07	170.5	0.43
SA III	30.2	0.29	168	6.7	1.52	0.058	0.066	166.6	0.46

### Content uniformity of the films:

The content uniformity and drug content of the each film was done by dissolved in 10 ml of double distilled water by stirring on a magnetic stirrer for 4 h after complete solubilization, filtered through Whatman filter paper. The filtrate is analyzed UV spectrometrically at 274 nm using a blank film solution as reference sample.

### Measurement of mucoadhesive strength:

The strength of the bond formed between the formulation and mucosa membrane excised from the porcine cheek pouch was determined using tensile experiments on a specially fabricated assembly slightly modified from the method described by Guptha *et al*^5^. The porcine cheek pouch was used as the model membrane and isotonic phosphate buffer pH 6.6 was used as the moistening fluid. The porcine cheek pouch was then stuck on to the inner surface of the Petri dish using suitable glue such that mucosal surface faces upwards. Then the phosphate buffer pH 6.6 was added in to Petri dish such that the buffer is contacted with the mucosal membrane. Two sides of the balance were made equal before the study, by keeping a 5 g weight on the left side. A Petri dish containing mucosal membrane was kept below the right hand set up of the balance. The test dummy films were stuck on to a lower flat side of hanging glass assembly. The surface of the mucosa was blotted with Whatman filter paper. 25 μl of IBP pH 6.6 was added to the mucosal surface. Five grams weight from the left pan was removed. This lowered the glass assembly along with film over the membrane with weight of 5 g. This was kept undisturbed for 3 min. Then the weights on the left hand side were slowly added till the film just separated from the membrane surface. The excess weight on the left pan i.e. total weight minus 5 g was taken as adhesive strength, the following parameters were calculated, force of adhesion (N) = bioadhesive strength × 9.81/1000 and bond strength (N/m^2^) =force of adhesion (N)/surface area (m^2^).

### Measurement of mucoadhesive time:

The mucoadhesive performance of the buccal films was evaluated using porcine buccal tissue. The time for film to detach from the porcine buccal tissue in a well-stirred beaker were used to assess the mucoadhesive performance. The fresh cut porcine buccal tissue was fixed on the side of the beaker with glue. Before addition of the buffer, the films were attached to porcine buccal tissue by applying light force (approximately 0.5N) with fingertip for 20 s. The beaker was then filled with 800 ml phosphate buffer and kept at 37°. A stirring rate of 150 rpm were used to simulate buccal and saliva movement. The attachment of films was monitored until drug release time. The time for the film to detach from the porcine buccal tissue was recorded as the mucoadhesion time 6.

### Measurement of water vapor through films:

From each blank film of SA I, SA II and SA III was taken for test. In empty vial approximately 1 g of dried a hydrous calcium chloride was taken, and at the neck of the vial blank films were pasted. And these vials were initially weighed and they were kept in desiccators, which contains saturated solution of potassium chloride, to maintain 75±5 %RH. Desiccator was tightly closed. And the vials were weighed after 1, 2, 3, 4, 5, 6 and 7^th^ day 7.

### Measurement of folding endurance of the films:

A modified USP Tablet disintegration tester was used for determining the folding endurance of the films. It consists of a fixed and a movable jaw that could be moved up and down at the rate of 28 strokes per min. The distance between the two jaws at their farthest and closest was 6 and 0.5 cm, respectively. The film (8 cm in length) was clamped between the two jaws in such a way that the jaws were at their closest, the film bent across its middle and when at their farthest, the film was in a stretched condition. Thus for every stroke of the movable jaw, the film went through one cycle of bending and stretching. The folding endurance is expressed as the number of strokes required to either break or develop visible cracks on the film. The test was conducted 1 h equating 1680 strokes 8.

### Measurement of Tensile strength of the films:

Tensile strength of the film is total weight, which is necessary to break or rupture the films and this was done by a device has rectangular frame with two plates made up of Plexiglas. The one plate is in the front and is the movable part of the device and can be pulled by loading weights on the string, which is connected to the movable plate. The testing procedure was performed as follows; the 1×2 cm^2^ films were fixed between the stationary and movable plate. The force needed to fracture the films was determined by measuring the total weight loaded in the string. The weight corresponds to break the films were taken as tensile strength and valves are given in Table 2^9^.

### Measurement of surface pH of the mucoadhesive films:

The mucoadhesive films were allowed in contact with 1 ml of distilled water. The surface pH was noted by bringing a combined glass electrode near the surface of films and allowing to equilibrate for 1 min 10.

### *Ex vivo* permeation through porcine buccal mucosa:

From the local slaughterhouse the buccal mucosa was collected and immediately transported to the laboratory in cold normal saline solution. The buccal mucosa, with a part of submucosa, was carefully separated from fat and muscles using scalpel. Then buccal epithelium was isolated from the underlying tissue. The buccal epithelium was used within 2 h upon removal. The modified Franz diffusion cell was used to permeation studies, it consists of two compartments, one is donor compartment and another is receptor compartment of 18 ml capacity and having 0.785 cm^2^ effective diffusion area. The receptor compartment was covered with water jacket to maintain temperature 37°. The separated buccal epithelium was mounted between two chambers and in receptor chamber PBS pH 7.4 was filled and buccal epithelium was allowed to stabilization for the period of 1 h. After stabilization of buccal epithelium, the film was kept on buccal epithelium and periodically samples were withdrawn and same volume fresh medium was replaced. The aliquots were analyzed spectrophotometrically.

### Characterization of the bilayered films:

The surface morphology and cross sections of the films were examined by Scanning Electron Microscopy. The dried films were coated under argon atmosphere with gold-palladium to achieve a film of 20 mm thickness and then observed under a scanning electron microscope (Jeol JSM-840. Japan).

## RESULTS AND DISCUSSION

**Fig. 1:**
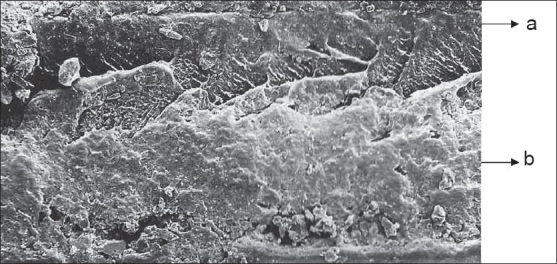
SEM showing a cross section of a mucoadhesive bilaminated film. Cross section of a mucoadhesive bilaminated film (a) ethyl cellulose/dbp backing layer and (b) sodium alginate/glycerin mucoadhesive layer.

The main goal of this work was to develop a new sodium alginate/ethylcellulose mucoadhesive bilaminated film, consisting of a drug containing mucoadhesive layer and a drug free non-adhesive protective layer. The bilaminated films developed by us were flexible and having suitable toughness. On the other hand, it is important to mention that the sodium alginate (hydrophilic) was easily laminated on to ethylcellulose (hydrophobic) and that a perfect binding between the mucoadhesive and backing layers was achieved, as is clearly demonstrated by SEM of film cross sections (fig. 1).

The bioadhesive strength exhibited by films was satisfactory for maintaining them in oral cavity. This aspect was further confirmed by measurement of mucoadhesive time. The important characteristics of different mucoadhesive films are given in Table 2.

During mucoadhesive time studies, no films were dropped from the porcine buccal tissue within 48 h of experiment time. In folding endurance test no films developed any visible cracks or breaks, thus showing good folding endurance. The surface pH of the films was determined in order to investigate the possibility of any side effects, in the oral cavity.

**Fig. 2:**
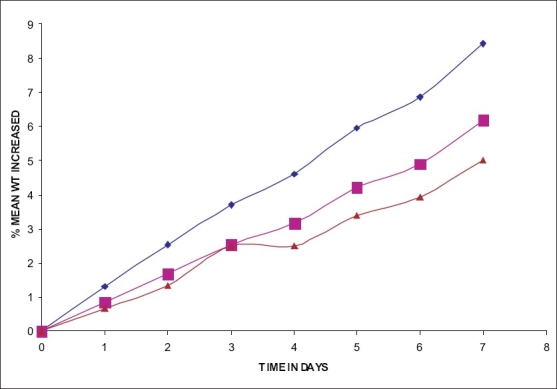
% Mean wt increased vs. time graph water vapor transmission study. The percent mean weight increased against time for SA I (—■—), SA II (—♦—) and for SA III (—▲—).

**Fig. 3:**
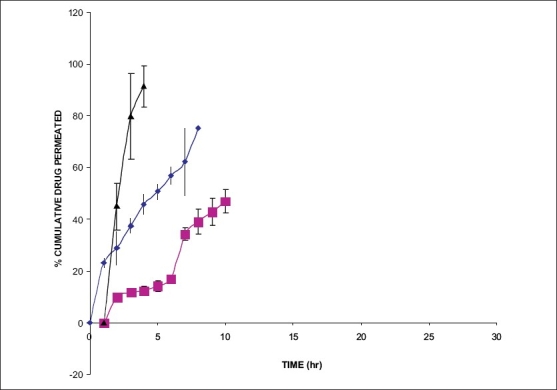
Plot of percentage drug permeated with time for SA films for *ex vivo* studies. *Ex vivo* cumulative permeation of atenolol from film SA I (—♦—), SA II (—■—) and for SA III (—▲—).

**Fig. 4:**
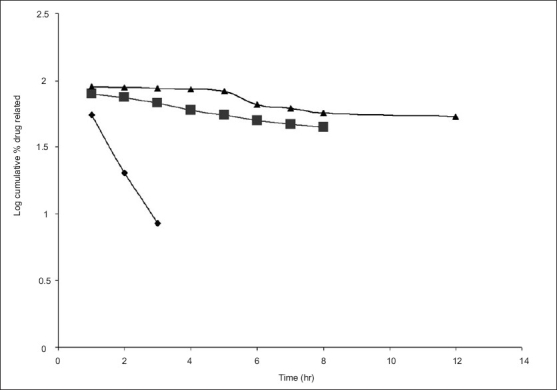
Plot of First order kinetic diffusion for SA films for *ex vivo* studies. Log cumulative atenolol retained against time for the film SA I (—♦—), SA II (—■—) and for SA III (—▲—).

**Fig. 5:**
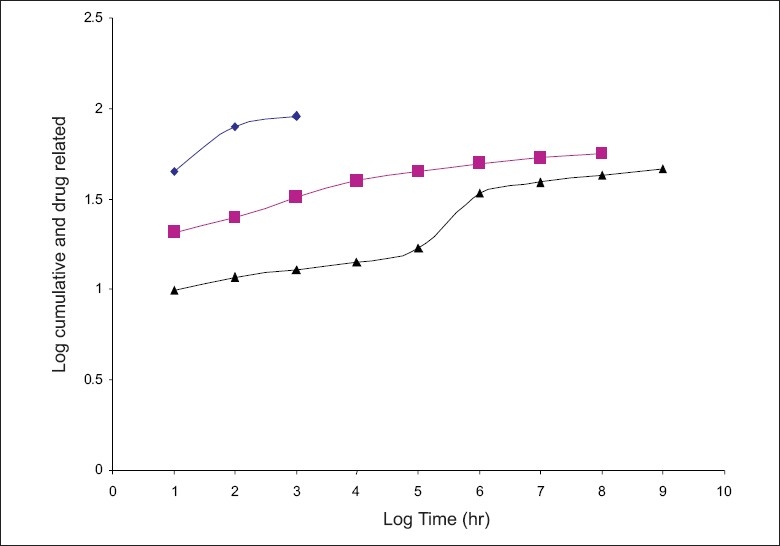
Double log plot for SA I, SA II and SAIII films. Log cumulative percent atenolol permeated against log time for SA I (–♦–), SA II (–■–) and for SA III (–▲–).

The hydration rates of the mucoadhesive films were shown in Table 2 and it suggested that the films containing more amount of carbopol 934P shows more hydration rate than other films, this would be due to more swelling of polymers and hold more amount of water in their network. A profile of water vapor transmission through various films was given in Table 2, with their permeability co efficient. The plots of flux vs. time (fig. 2) were straight line, which intersected the origin, indicating that there existed a steady state of permeation through the films. The release rate from different films shows that, release of drug decrease with increase in viscosity of the formulations. The release of drug from these films exhibits two phases. There is a initial burst effect is followed by the completion of a stable gel layer which in turn, controls the release of drug from the delivery system. As it can be seen in (fig. 3), film with only sodium alginate yielded a faster initial burst effect. The increased viscosity of formulation resulted in a corresponding decrease in the drug release 11 and it is also postulated that, with higher viscosity resulted in thicker gel layer formation 12. It appears that the incorporation of carbopol 934P decreased the drug release from the films, since carbopol is insoluble in water or simulated saliva, and swelling behavior of the carbopol is attributed to the uncharged –COOH group that get hydrated by forming hydrogen bonds with the imbibing water and, therefore, extending polymer chain 13. The drug release from the optimized film follows the apparent first order kinetics, since the plot of the log % drug remaining against time (fig. 4) gave a linear curve (R^2^ =0.9691) for SA II film revealing apparent first order kinetics. To ascertain the drug permeation follows the Fickian type or non-Fickian release, log cumulative amount of drug permeated vs log time plot (fig. 5) was plotted and The value of n (n=0.5125) was estimated by linear regression (R^2^ =0.9349), theses values indicates the drug permeation non-Fickian kinetics.

In conclusion, mucoadhesive films of atenolol were successfully developed. It exhibited well-controlled and delayed release pattern. This study concludes that, the addition of carbopol 934P increases the viscosity and swelling of films there by controls the release of drug and improves the mucoadhesive properties.
